# Prevalence and Associated Factors of Low Bone Mineral Density in the Femoral Neck and Total Hip in Axial Spondyloarthritis: Data from the CASTRO Cohort

**DOI:** 10.3390/jcm10122664

**Published:** 2021-06-17

**Authors:** Laura Bautista-Aguilar, Clementina López-Medina, Lourdes Ladehesa-Pineda, María del Carmen Ábalos-Aguilera, Desirée Ruiz-Vilchez, Juan Luis Garrido-Castro, Ignacio Gómez-García, María Ángeles Puche-Larrubia, Asunción Salmoral-Chamizo, Eduardo Collantes-Estévez, Alejandro Escudero-Contreras, Pilar Font-Ugalde

**Affiliations:** 1 Rheumatology Department, Reina Sofia University Hospital, 14004 Cordoba, Spain; laurabautistaag@gmail.com (L.B.-A.); lourdesladehesapineda@gmail.com (L.L.-P.); mc.abalos@outlook.com (M.d.C.Á.-A.); desiree.ruiz@imibic.org (D.R.-V.); ignaciogomgar@gmail.com (I.G.-G.); mangeles.puche@gmail.com (M.Á.P.-L.); asuncionsalmoral61@gmail.com (A.S.-C.); educollantes@yahoo.es (E.C.-E.); alexcudero2@gmail.com (A.E.-C.); fougp@hotmail.com (P.F.-U.); 2 GC05 Group, Maimonides Institute of Biomedical Research of Cordoba, 14004 Cordoba, Spain; 3 Medical and Surgical Sciences Department, University of Cordoba, 14004 Cordoba, Spain; cc0juanl@uco.es

**Keywords:** axial spondyloarthritis, bone mineral density, osteopenia

## Abstract

Studies on osteoporosis in axial spondyloarthritis (axSpA) have focused on the lumbar segment, and few studies have assessed bone mineral density (BMD) in the hip and femoral neck in these patients. The aim of this study was to evaluate the prevalence of low BMD and osteopenia in the total hip or femoral neck and the factors associated with these conditions in axSpA patients. This was a single-centre, observational, cross-sectional study among consecutive patients with axSpA according to the ASAS criteria from the CASTRO registry. All patients underwent total hip and femoral neck DXA BMD measurements. Low BMD was defined as a Z-score less than −1, and osteopenia was defined as a T-score less than −1. Multivariate logistic and generalised linear regressions were used to evaluate factors independently associated with low BMD and osteopenia in the hip or femoral neck and those associated with variability in BMD, respectively. A total of 117 patients were included, among which 30.8% were female and the mean age was 45 years. A total of 36.0% of patients had low BMD (28.1% in the total hip and 27.4% in the femoral neck), and 56.0% of patients had osteopenia (44.7% in the total hip and 53.8% in the femoral neck). A multivariate logistic regression showed that age, radiographic sacroiliitis and ASAS-HI were independently associated with low BMD in the total hip or femoral neck. Factors that were independently associated with osteopenia were Body Mass Index, disease duration, radiographic sacroiliitis and ASAS-HI. In conclusion, 36% of the patients with axSpA had low BMD in the total hip or femoral neck. A younger age and radiographic sacroiliitis were the most important factors associated with decreased BMD.

## 1. Introduction

Spondyloarthritis (SpA) is a heterogeneous group of rheumatic diseases that involve the axial skeleton and peripheral joints [1]. Patients with predominantly axial symptoms are generally classified as having axial SpA (axSpA), and those with predominantly peripheral symptoms are typically diagnosed with peripheral SpA (pSpA) [2]. Axial SpA is characterised by inflammatory back pain, vertebral fusion in some cases and restricted spinal mobility. During the clinical course of the disease, patients with axSpA may also suffer from other clinical disorders, known as comorbidities. Osteoporosis is the most frequent comorbidity in these patients, with a global prevalence of 13.4% [3].

Bone remodelling is altered in axSpA patients, as inflammation causes osteoproliferation in cortical areas of the vertebrae and the loss of trabecular bone in vertebral bodies. As a consequence, low bone mass and biomechanical alteration of the spine produce an increased risk of vertebral fractures [4,5]. SpA has been associated with bone loss not only in the spine but also in the hip [6]. Factors associated with a decrease in bone mineral density (BMD) in these patients are systemic inflammation, which is evaluated by C-reactive protein (CRP) levels or the erythrocyte sedimentation rate [4], and local inflammation, which is diagnosed by bone marrow oedema on magnetic resonance imaging (MRI) of the spine [5]. In addition, impaired back mobility and spinal ankylosis have been associated with low BMD [7].

Most studies evaluating BMD in SpA have focused on the lumbar spine. However, lumbar BMD in axSpA patients can be overestimated due to the presence of syndesmophytes or other structural lesions, such as ankylosis of the posterior ligament and periosteal bone formation [8]. In addition, inflammation in these patients has a direct effect on the trabecular bone of the vertebrae but not on the cortical bone. Consequently, lumbar dual-energy X-ray absorptiometry (DXA) has several limitations in the evaluation of BMD [9]. For this reason, the European League Against Rheumatism (EULAR) taskforce provided recommendations on the evaluation of BMD in SpA patients. According to these guidelines, in patients with syndesmophytes in the lumbar spine on conventional radiography, BMD should be assessed using hip DXA supplemented with either DXA of the lateral projection of the spine or quantitative computed tomography (QCT) of the spine [10]. However, despite these recommendations, the majority of studies evaluating osteoporosis in axSpA patients have focused on the lumbar segment, and very few published studies have evaluated BMD in the hip and femoral neck in these patients.

Therefore, we conducted this study with the aim of assessing the prevalence of low BMD (i.e., Z-score less than −1) and osteopenia (i.e., T-score less than −1) and the factors associated with these conditions (in both the femoral neck and total hip) in axSpA patients.

## 2. Materials and Methods

### 2.1. Study Population

This was a single-centre, observational, cross-sectional study of 117 consecutive patients with axSpA according to the Assessment of Spondylitis International Society (ASAS) criteria from the Córdoba Axial Spondyloarthritis Task Force, Registry and Outcomes (CASTRO). The CASTRO registry includes 182 axSpA patients; however, 65 patients were excluded from this specific analysis for several reasons. Excluded patients were those who were (a) missing data for DXA of the total hip and femoral neck (*n* = 55); (b) receiving treatment with drugs that could interfere with bone metabolism (bisphosphonates, strontium ranelate, selective oestrogenic receptor modulators, calcitonin, hormone therapy, denosumab or teriparatide) (*n* = 4); (c) suffering from metabolic bone diseases (hyperthyroidism, hypercortisolism, hyperparathyroidism, malabsorption syndrome, Paget’s disease or malignant tumours) (*n* = 1); (d) receiving treatment that could cause osteoporosis (corticosteroids, acenocoumarol, heparin or anticonvulsants) (*n* = 2); and (e) on biological disease-modifying drugs (bDMARDs) (*n* = 3). This study was approved by the Ethics Committee at the Reina Sofia University Hospital (protocol code PI-0139-2017), and all the patients signed informed consent forms for inclusion. 

### 2.2. Data Collection

Data were collected during medical visits to the Rheumatology Unit of the Reina Sofia University Hospital in Cordoba, Spain. The following data were collected:

- Demographic data: Age, sex, Body Mass Index (BMI) and smoking status were obtained.
- Clinical data: Disease duration, diagnostic delay, and previous history of arthritis, enthesitis, dactylitis, uveitis, psoriasis, inflammatory bowel disease (IBD) and uveitis were identified. Radiographs of the cervical spine, lumbar spine and sacroiliac joints were obtained at the time of the BMD assessment. Lateral views of the cervical and lumbar spine were scored according to the modified Stoke Ankylosing Spondylitis Spinal Score (mSASSS index) [11]. Sacroiliitis was scored according to the modified New York criteria [12]. Sacroiliitis and mSASSS were scored by two trained rheumatologists who were blinded to patient characteristics. The intraclass correlation coefficient score for agreement between the readers was 0.99 for the total mSASSS, and the mean value of the two readers was used. The kappa score for agreement between the two readers for the modified New York criteria was 0.76. In cases of disagreement, the evaluation from the senior reader was used.
- Blood test: HLA-B27 antigen and CRP levels were reported.
- Treatments: Data on the use of non-steroidal anti-inflammatory drugs (NSAIDs), corticosteroid intake and vitamin D supplementation were collected.
- Disease activity and function: The self-administered questionnaires from the Bath Ankylosing Spondylitis Disease Activity Index (BASDAI) [13], Bath Ankylosing Spondylitis Functional Index (BASFI) [14] and ASAS-endorsed Disease Activity Score (ASDAS) [15] were used. In addition, CRP levels (mg/L) were recorded retrospectively once, twice or three times during the 5 years prior to the study and at the time of the study to evaluate the persistence of inflammation. A patient was considered to have persistent inflammation if they had increased CRP levels (>10 mg/L) in at least 50% of the measurements in the previous 5 years.
- Bone mineral density (BMD): All participants underwent DXA BMD measurement of the total hip and femoral neck using a dual X-ray absorptiometry (DXA) LUNAR DPX 8548 BX-1 L densitometer (coefficient of variation < 1%). All measurements were made by the same operator. World Health Organization (WHO) criteria were used for the diagnosis of osteopenia (i.e., T-score less than −1), osteoporosis (i.e., T-score less than −2.5), low BMD (Z-score less than −1) and very low BMD (Z-score less than −2) [16].
- Spinal mobility was studied using both the total University of Cordoba Ankylosing Spondylitis Metrology Index (UCOASMI) and the individual items of the UCOASMI, which is a composite index based on an automated system that generates a cervical and vertebral mobility score from serial kinematic measurements [17,18,19]. Among other measures, it evaluates lumbar anterior flexion, lateral flexion and rotation in grades. The total UCOASMI score ranges from 0 to 10 (from better to worse mobility). The motion video-capture system consists of 11 reflective markers placed in anatomical points, four cameras and specific software (UCOTrack). Markers are attached in less than 2 min. The patient must then perform specific movements, such as flexion, extension and rotation. The software interprets the images and generates summary measures that are included in the index: cervical frontal flexion, cervical rotation, frontal spinal flexion, shoulder–hip lateral angle and trunk rotation.

### 2.3. Statistical Analysis

A descriptive analysis of the variables was conducted. Absolute and relative frequencies were calculated for the qualitative variables, and the mean and standard deviation (SD) were determined for continuous values. 

Univariate and multivariate logistic regression models using a backward stepwise procedure were conducted to evaluate sociodemographic, clinical and disease activity factors associated with low BMD (i.e., Z-score less than −1) in the total hip or femoral neck. Variables with a *p*-value < 0.20 in the univariate analysis were subjected to a multivariate analysis, and the degree of association was expressed as the odds ratio (OR) and 95% confidence interval. Interaction and confounding factors were tested. The same analysis was conducted to evaluate factors associated with osteopenia (i.e., T-score less than −1) in the total hip or femoral neck. 

Finally, to determine the factors independently associated with BMD, two separate univariate and multivariate generalised linear models (one for the femoral neck and a second one for the total hip) were conducted. Multicollinearity, homogeneity of variance and normality of residuals were tested.

All comparisons were bilateral, and *p* < 0.05 was considered a significant result. Data were analysed using R Studio 1.3.1073 © (www.rstudio.com, accessed on 14 April 2021).

## 3. Results

Among the 117 patients included, 30.8% were female, and the mean age was 45 years old. A total of 36.0% of patients had low BMD (28.1% in the total hip and 27.4% in the femoral neck). In addition, a total of 56.0% of patients had osteopenia (44.7% in the total hip and 53.8% in the femoral neck). The demographic and clinical characteristics of the patients are summarised in Table 1.

### 3.1. Factors Associated with Low BMD in the Total Hip or Femoral Neck

**Figure 1:**
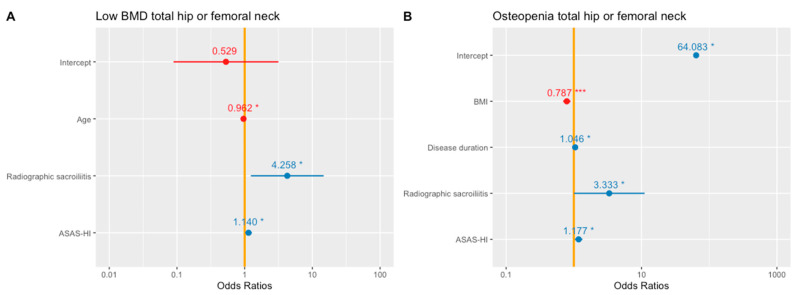
Multivariate logistic regression to evaluate the factors associated with low BMD (**A**) and osteopenia (**B**) in the total hip or femoral neck. ASAS-HI: ASAS Health Index; BMI: Body Mass Index. *, *p*-value < 0.05; ***, *p*-value < 0.001.

Univariate logistic regression (Table 2) showed no significant association between low BMD in the total hip or femoral neck and the other covariates. However, the multivariate logistic regression (Figure 1A) showed that age (OR 0.96, 95% CI 0.93–0.99), radiographic sacroiliitis (OR 4.26, 95% CI 1.33–16.34) and higher ASAS-HI scores (OR 1.14, 95% CI 1.02–1.28) were independently associated with low BMD of the total hip or femoral neck.

### 3.2. Factors Associated with Osteopenia in the Total Hip or Femoral Neck

The univariate logistic regression (Table 3) showed a significant association between osteopenia in the total hip or femoral neck and age (OR 1.03, 95% CI 1.01–1.07), BMI (OR 0.86, 95% CI 0.77–0.94), disease duration (OR 1.04, 95% CI 1.01–1.07), ASAS-HI (OR 1.10, 95% CI 1.00–1.23) and BASFI (OR 1.19, 95% CI 1.03–1.40). In addition, the multivariate logistic regression (Figure 1B) showed that BMI (OR 0.79, 95% CI 0.69–0.89), disease duration (OR 1.05, 95% CI 1.01–1.09), radiographic sacroiliitis (OR 3.33, 95% CI 1.03–11.81) and higher ASAS-HI scores (OR 1.18, 95% CI 1.04–1.35) were independently associated with osteopenia in the total hip or femoral neck.

### 3.3. Factors Associated with Total Hip BMD

**Figure 2:**
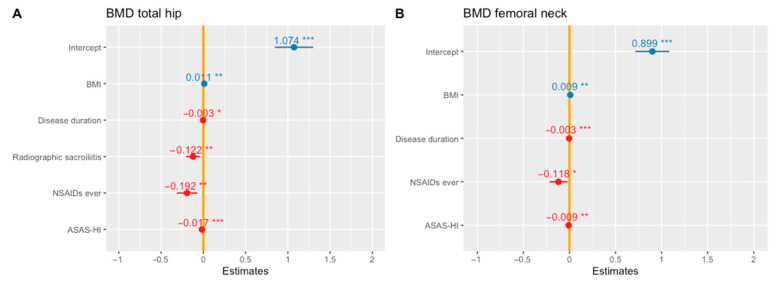
Multivariate generalised linear regressions to evaluate factors associated with bone mineral density in the total hip (**A**) and femoral neck (**B**). ASAS-HI: ASAS Health Index; BMI: Body Mass Index; NSAIDs: Non-steroidal anti-inflammatory drugs. *, *p*-value < 0.05; **, *p*-value < 0.01; ***, *p*-value < 0.001.

A univariate generalised linear regression analysis was performed to determine factors associated with the total hip BMD in axSpA patients (Table 4), and the results showed no significant effect of age, BMI, disease duration, HLA B-27, UCOASMI, mSASSS or persistent CRP elevation. However, significant associations were found between the total hip BMD and female sex (*β* −0.082, 95% CI −0.154 to −0.011), radiographic sacroiliitis (*β* −0.097, 95% CI −0.180 to −0.014), psoriasis (*β* 0.111, 95% CI 0.012–0.211), lumbar flexion (*β* 0.002, 95% CI 0.000–0.004), previous or current intake of NSAIDs (*β* −0.156, 95% CI −0.277 to −0.034), ASAS-HI (*β* −0.014, 95% CI −0.022 to −0.005), BASDAI (*β* −0.016, 95% CI −0.031 to 0.001) and BASFI (*β* −0.018, 95% CI −0.031 to −0.006). A multivariate generalised regression analysis (Figure 2A) showed that BMI, disease duration, radiographic sacroiliitis, previous or current use of NSAIDs and ASAS-HI were independently associated with total hip BMD.

### 3.4. Factors Associated with Femoral Neck BMD

The univariate generalised linear regression analysis (Table 5) revealed associations between the femoral neck BMD and age (*β* −0.004, 95% CI −0.006 to −0.001), female sex (*β* −0.063, 95% CI −0.120 to −0.006), disease duration (*β* −0.003, 95% CI −0.005 to −0.001), lumbar flexion (*β* 0.001, 95% CI 0.000 to 0.002), ASAS-HI (*β* −0.010, 95% CI −0.017 to −0.003) and BASFI (*β* −0.016, 95% CI −0.026 to −0.006). 

The multivariate generalised linear regression analysis showed that BMI, disease duration, NSAID use and ASAS-HI were independently associated with femoral neck BMD (Figure 2B). 

## 4. Discussion

Our study showed that 36.0% of patients with axSpA had low BMD (28.1% in the total hip and 27.4% in the femoral neck), and a total of 56.0% of patients had osteopenia (44.7% in the total hip and 53.8% in the femoral neck). The prevalence of low BMD in our population is similar to that published in the scientific literature [6,7,20,21]. However, it should be noted that lumbar DEXA was not considered in this study due to the influence of lumbar syndesmophytes and ankylosis on BMD measurements [22]. According to the EULAR taskforce, in patients with axSpA without syndesmophytes in the lumbar spine on conventional radiography, osteoporosis should be assessed using hip DXA and anterior–posterior spine DXA; however, in patients with syndesmophytes in the lumbar spine on conventional radiography, osteoporosis should be assessed using hip DXA supplemented with either spine DXA (lateral projection) or a QCT of the spine [10]. QCT information was not available for this CASTRO cohort, and many of these patients had syndesmophytes and structural damage at the lumbar level. In addition, we found that BMD in the lumbar spine was higher than that in the hip, which may be explained by the presence of new bone formation. For these reasons, we focused our analysis on hip DXA, since very few published studies have evaluated hip and femoral neck BMD in these patients. 

Traditional risk factors for low BMD in the general population are female sex, increasing age, low Body Mass Index, menopause and the presence of chronic inflammatory diseases [6,23]. Interestingly, we found that a younger age was independently associated with low BMD in the total hip or femoral neck in patients with axSpA. Therefore, it seems that mainly younger axSpA patients (both males and females) are suffering from low BMD, in contrast to primary osteoporosis, which is mostly found in older females. In the univariate analysis, low BMI was independently associated with the presence of osteopenia in the total hip and femoral neck. The reason for this association is that stress on the bone wall increases osteoblastic activity, and therefore, bone mass increases. Stress on the bone wall is mainly influenced by physical activity and other factors, such as muscle mass and fat tissue, due to the pull of gravity acting on the weight of soft tissues (fat, muscle). Thus, low BMI is associated with low BMD, less soft tissue and muscle weakness [24]. In addition, osteopenia was independently associated with a longer disease duration in our study. It has been reported that patients with long-standing axSpA have a higher risk of muscle loss because of reduced physical activity, immobilisation and inflammation [25,26]. Consistent with these reports, we found that both low BMD and osteopenia in either the total hip or femoral neck were associated with radiographic sacroiliitis, which may, in part, be a reflection of patients with a more severe disease and lower mobility, although no direct association was found with the UCOASMI. Inflammation imaged by an MRI, and systemic inflammation evaluated by C-reactive protein levels or the erythrocyte sedimentation rate have been proposed as factors associated with BMD loss [4]. However, we did not find an association with CRP or with ASDAS. 

Due to the direct effect of inflammation on BMD, the use of NSAIDs and TNF blockers has been described as a protective factor against BMD loss [5,27]. The positive effect of NSAIDs may be explained by a direct effect on bone metabolism. NSAIDs might slow new bone formation in the spine and prevent heterotopic ossification by acting on biological factors such as bone morphogenetic proteins, metalloproteinases and PG receptor genes. In addition, NSAIDs may have an indirect effect through an increase in physical activity due to pain relief and the control of inflammation [5,28]. The effect of TNF-alpha blockers on increased BMD is attributed to its key role in bone resorption and formation. Osteoclast activity is enhanced by TNF-alpha, which also inhibits osteoclast apoptosis. In addition, excess TNF-alpha leads to the inhibition of the bone formation process [5,29]. Thus, the use of TNF-alpha blockers has demonstrated a similar effect on bone remodelling markers to that of antiresorptive drugs [5,30]. In fact, a metanalysis published by Haroon et al. demonstrated a significant improvement in BMD in the total hip after 1 and 2 years of follow-up in patients on TNF-alpha blockers [31]. In our study, we excluded patients under bDMARD therapy precisely to avoid bias in BMD measurements in patients on this type of drug. On the other hand, we found an inverse association between NSAID use and total hip BMD. This may be explained by the higher utilisation of NSAIDs in patients with a more severe disease, as has also been described in previous studies [32]. Interestingly, we found very low vitamin D levels in this study population, which may be explained by the fact that vitamin D levels were determined for the first time during this study visit. This makes us reflect on the necessity of systematically evaluating bone mineral metabolism in SpA patients.

Few studies have assessed the association between BMD and ASAS-HI, since this is a relatively recent metric that evaluates the health status in patients with SpA. Our results demonstrate that osteopenia or osteoporosis and low BMD in the hip and femoral neck are associated with poorer ASAS-HI scores, which may be driven by reduced function and mobility [33,34,35]. 

Our study has strengths and limitations. One limitation is the cross-sectional nature of the study, which prevents us from evaluating the causal association between osteopenia or osteoporosis and clinical factors. Another limitation is that this is a single-centre study. One strength of this study is that we removed patients on bDMARDs to avoid the effect of TNF-alpha blockers on BMD measurements. In addition, mobility was evaluated using an automated system to avoid intra-observer variability. Finally, X-rays (sacroiliitis and mSASSS) were scored by two rheumatologists to obtain reliable data.

## 5. Conclusions

In conclusion, our study showed that 36.0% of patients with axSpA had low BMD in the total hip or femoral neck. A younger age and radiographic sacroiliitis were the most important factors associated with this decrease in BMD. Prospective studies focused on hip BMD are needed to better understand bone metabolism in axSpA patients.

## Figures and Tables

**Table 1:** Descriptive data of the included population.

	Total *n* = 117
Sex (female)	36 (30.8%)
Age, mean (SD)	45.4 (12.1)
BMI, mean (SD)	26.6 (4.1)
Caucasian ethnicity	117 (100%)
Current smoking	45 (38.5%)
Disease duration, mean (SD)	19.2 (13.9)
Diagnosis delay, mean (SD)	5.2 (6.3)
Radiographic sacroiliitis	94/116 (81.0%)
HLA-B27 positive	91/115 (79.1%)
Arthritis (ever)	25 (21.4%)
Enthesitis (ever)	15/113 (13.3%)
Dactylitis (ever)	8 (6.8%)
Psoriasis	14 (12.0%)
Uveitis (ever)	24 (20.5%)
IBD	4/112 (3.6%)
CRP, mean (SD)	6.5 (10.8)
ASDAS-CRP, mean (SD)	2.4 (0.9)
Persistent CRP	43 (36.8%)
NSAIDs (ever)	106/115 (92.2%)
Vitamin D supplementation (ever)	6 (5.1%)
UCOASMI, mean (SD)	4.2 (1.7)
Lumbar flexion, mean (SD)	58.7 (22.0)
Total mSASSS, mean (SD)	13.9 (16.3)
ASAS-HI, mean (SD)	4.8 (4.0)
BASDAI, mean (SD)	3.6 (2.1)
BASFI, mean (SD)	3.1 (2.6)
Total hip BMD (g/cm^2^)	0.98 (0.18)
Femoral neck BMD (g/cm^2^)	0.92 (0.15)
Lumbar BMD (g/cm^2^)	1.11 (0.19)
Total hip BMD T-score less than −1 (osteopenia)	51/114 (44.7%)
Total hip BMD T-score less than −2.5 (osteoporosis)	2/114 (1.8%)
Total hip BMD Z-score less than −1 (low BMD)	32/114 (28.1%)
Total hip BMD Z-score less than −2 (very low BMD)	6/114 (5.3%)
Femoral neck BMD T-score less than −1 (osteopenia)	63 (53.8%)
Femoral neck BMD T-score less than −2.5 (osteoporosis)	4 (3.4%)
Femoral neck BMD Z-score less than −1 (low BMD)	32 (27.4%)
Femoral neck BMD Z-score less than −2 (very low BMD)	4 (3.4%)
Total hip or femoral neck T-score less than −1 (osteopenia)	65/116 (56.0%)
Total hip or femoral neck Z-score less than −1 (low BMD)	41/114 (36.0%)
Lumbar BMD T-score less than −1 (osteopenia)	57 (48.7%)
Lumbar BMD T-score less than −2.5 (osteoporosis)	11 (9.4%)
Lumbar BMD Z-score less than −1 (low BMD)	55 (47.0%)
Lumbar BMD Z-score less than −2 (very low BMD)	18 (15.4%)
Current vitamin D level (ng/mL), mean (SD)	17.1 (9.8)

ASAS-HI: ASAS Health Index; ASDAS: ASAS-endorsed Disease Activity Score; BASDAI: Bath Ankylosing Spondylitis Disease Activity Index; BASFI: Bath Ankylosing Spondylitis Functional Index; BMD: bone mineral density; BMI: Body Mass Index; CRP: C-reactive protein; IBD: inflammatory bowel disease; mSASSS: modified Stoke Ankylosing Spondylitis Spinal Score; NSAIDs: non-steroidal anti-inflammatory drugs; SD: standard deviation; UCOASMI: University of Cordoba Ankylosing Spondylitis Metrology Index.

**Table 2:** Univariate logistic regression to evaluate the factors associated with low BMD in the total hip or femoral neck.

	Low BMD in the Total Hip or Femoral Neck			
	Yes = 41 *n* (%)	No = 73 *n* (%)	OR (95% CI)	*p*-Value
Age, mean (SD)	43.0 (12.2)	46.2 (11.9)	0.98 (0.94–1.01)	0.168
Sex (female)	12 (29.3%)	22 (30.1%)	0.96 (0.41–2.20)	0.923
BMI, mean (SD)	26.2 (4.7)	26.9 (3.9)	0.96 (0.87–1.06)	0.423
Current smoking	20 (48.8%)	23 (31.5%)	2.07 (0.94–4.58)	0.070
Disease duration, mean (SD)	18.9 (13.7)	19.3 (14.2)	0.99 (0.97–1.03)	0.882
HLA-B27	28/39 (71.8%)	61 (83.6%)	0.50 (0.20–1.28)	0.146
Radiographic sacroiliitis	36 (87.8%)	55/72 (76.4%)	2.23 (0.80–7.25)	0.147
Psoriasis	5 (12.2%)	9 (12.3%)	0.99 (0.29–3.09)	0.983
IBD	3/38 (7.9%)	1/71 (1.4%)	6.00 (0.74–123.72)	0.127
ASDAS-CRP, mean (SD)	2.5 (1.0)	2.3 (0.9)	1.23 (0.82–1.85)	0.318
Persistent CRP	16 (39.0%)	25 (34.2%)	1.23 (0.55–2.71)	0.610
Total mSASSS, mean (SD)	13.2 (16.3)	14.1 (16.6)	0.99 (0.97–1.02)	0.780
UCOASMI, mean (SD)	4.1 (1.5)	4.2 (1.8)	0.95 (0.74–1.21)	0.695
Lumbar flexion, mean (SD)	57.5 (21.8)	59.6 (22.7)	0.99 (0.98–1.01)	0.656
NSAIDs (ever)	36/40 (90.0%)	67/72 (93.1%)	0.67 (0.17–2.86)	0.571
ASAS-HI, mean (SD)	5.4 (4.2)	4.2 (3.8)	1.07 (0.97–1.19)	0.160
BASDAI, mean (SD)	3.8 (2.4)	3.4 (2.0)	1.09 (0.91–1.30	0.352
BASFI, mean (SD)	3.6 (2.6)	2.7 (2.5)	1.13 (0.98–1.32)	0.105

ASAS-HI: ASAS Health Index; ASDAS: ASAS-endorsed Disease Activity Score; BASDAI: Bath Ankylosing Spondylitis Disease Activity Index; BASFI: Bath Ankylosing Spondylitis Functional Index; BMD: bone mineral density; BMI: Body Mass Index; CRP: C-reactive protein; IBD: inflammatory bowel disease; mSASSS: modified Stoke Ankylosing Spondylitis Spinal Score; NSAIDs: non-steroidal anti-inflammatory drugs; SD: standard deviation; UCOASMI: University of Cordoba Ankylosing Spondylitis Metrology Index.

**Table 3:** Univariate logistic regression to evaluate the factors associated with osteopenia in the total hip or femoral neck.

	Osteopenia in the Total Hip or Femoral Neck			
	Yes = 65 *n* (%)	No = 51 *n* (%)	OR (95% CI)	*p*-Value
Age, mean (SD)	47.6 (12.8)	42.5 (10.5)	1.03 (1.01–1.07)	0.026
Sex (female)	20 (30.7%)	15 (29.4%)	1.07 (0.48–2.4)	0.874
BMI, mean (SD)	25.6 (3.8)	28.0 (4.3)	0.86 (0.77–0.94)	0.003
Current smoking	26 (40.0%)	18 (35.3%)	1.22 (0.57–2.63)	0.604
Disease duration, mean (SD)	22.1 (15.2)	15.4 (11.0)	1.04 (1.01–1.07)	0.013
HLA-B27	48/63 (76.2%)	42 (82.4%)	0.69 (0.26–1.70)	0.424
Radiographic sacroiliitis	56 (86.2%)	37/50 (74.0%)	2.19 (0.86–5.80)	0.105
Psoriasis	7 (10.8%)	7 (13.7%)	0.76 (0.24–2.37)	0.628
IBD	3/61 (4.9%)	1/50 (2.0%)	2.53 (0.31–52.13)	0.427
ASDAS-CRP, mean (SD)	2.5 (1.0)	2.3 (0.9)	1.21 (0.82–1.81)	0.346
Persistent CRP	24 (36.9%)	19 (37.3%)	0.99 (0.46–2.11)	0.971
Total mSASSS, mean (SD)	14.8 (16.7)	12.4 (15.9)	1.01 (0.99–1.04)	0.434
UCOASMI, mean (SD)	4.3 (1.7)	4.1 (1.7)	1.06 (0.84–1.35)	0.621
Lumbar flexion, mean (SD)	54.8 (20.4)	63.4 (23.4)	0.98 (0.96–1.00)	0.057
NSAIDs (ever)	59/63 (93.7%)	46/51 (90.2%)	1.60 (0.40–6.80)	0.500
ASAS-HI, mean (SD)	5.4 (4.1)	3.9 (3.7)	1.10 (1.00–1.23)	0.047
BASDAI, mean (SD)	3.7 (2.2)	3.4 (2.1)	1.08 (0.91–1.29)	0.369
BASFI, mean (SD)	3.6 (2.6)	2.5 (2.4)	1.19 (1.03–1.40)	0.024

ASAS-HI: ASAS Health Index; ASDAS: ASAS-endorsed Disease Activity Score; BASDAI: Bath Ankylosing Spondylitis Disease Activity Index; BASFI: Bath Ankylosing Spondylitis Functional Index; BMD: bone mineral density; BMI: Body Mass Index; CRP: C-reactive protein; IBD: inflammatory bowel disease; mSASSS: modified Stoke Ankylosing Spondylitis Spinal Score; NSAIDs: non-steroidal anti-inflammatory drugs; SD: standard deviation; UCOASMI: University of Cordoba Ankylosing Spondylitis Metrology Index.

**Table 4:** Univariate generalised linear regression to evaluate the factors associated with total hip bone mineral density.

	Beta Coefficient (95% CI)	*p*-Value
Age	−0.003 (−0.005 to 0.000)	0.067
Sex (female)	−0.082 (−0.154 to −0.011)	0.026
BMI	0.008 (−0.001 to 0.016)	0.055
Current smoking	−0.013 (−0.081 to 0.056)	0.722
Disease duration	−0.002 (−0.004 to 0.001)	0.056
HLA-B27	0.013 (−0.071 to 0.097)	0.761
Radiographic sacroiliitis	−0.097 (−0.180 to −0.014)	0.024
Psoriasis	0.111 (0.012–0.211)	0.031
IBD	−0.095 (−0.277 to 0.087)	0.308
ASDAS-CRP	−0.034 (−0.069 to 0.001)	0.056
Persistent CRP	−0.001 (−0.069 to 0.071)	0.976
Total mSASSS	−0.001 (−0.003 to 0.001)	0.357
UCOASMI	−0.011 (−0.033 to 0.010)	0.308
Lumbar flexion	0.002 (0.000–0.004)	0.025
NSAIDs (ever)	−0.156 (−0.277 to −0.034)	0.013
ASAS-HI	−0.014 (−0.022 to −0.005)	0.002
BASDAI	−0.016 (−0.031 to 0.001)	0.046
BASFI	−0.018 (−0.031 to −0.006)	0.005

ASAS-HI: ASAS Health Index; ASDAS: ASAS-endorsed Disease Activity Score; BASDAI: Bath Ankylosing Spondylitis Disease Activity Index; BASFI: Bath Ankylosing Spondylitis Functional Index; BMD: bone mineral density; BMI: Body Mass Index; CRP: C-reactive protein; IBD: inflammatory bowel disease; mSASSS: modified Stoke Ankylosing Spondylitis Spinal Score; NSAIDs: non-steroidal anti-inflammatory drugs; SD: standard deviation; UCOASMI: University of Cordoba Ankylosing Spondylitis Metrology Index.

**Table 5:** Univariate generalised linear regression to evaluate factors associated with femoral neck bone mineral density.

	Beta Coefficient (95% CI)	*p*-Value
Age	−0.004 (−0.006 to −0.001)	0.002
Sex (female)	−0.063 (−0.120 to −0.006)	0.033
BMI	0.006 (−0.001 to 0.012)	0.052
Current smoking	−0.001 (−0.057 to 0.054)	0.960
Disease duration	−0.003 (−0.005 to −0.001)	0.006
HLA-B27	0.008 (−0.060 to 0.075)	0.820
Radiographic sacroiliitis	−0.056 (−0.125 to 0.013)	0.113
Psoriasis	0.030 (−0.053 to 0.113)	0.479
IBD	−0.085 (−0.233 to 0.064)	0.265
ASDAS-CRP	−0.022 (−0.051 to 0.006)	0.129
Persistent CRP	−0.009 (−0.065 to 0.047)	0.746
Total mSASSS	−0.001 (−0.002 to 0.001)	0.498
UCOASMI	−0.007 (−0.025 to 0.010)	0.399
Lumbar flexion	0.001 (0.000 to 0.002)	0.050
NSAIDs (ever)	−0.068 (−0.169 to 0.032)	0.187
ASAS-HI	−0.010 (−0.017 to −0.003)	0.004
BASDAI	−0.012 (−0.025 to 0.001)	0.061
BASFI	−0.016 (−0.026 to −0.006)	0.002

ASAS-HI: ASAS Health Index; ASDAS: ASAS-endorsed Disease Activity Score; BASDAI: Bath Ankylosing Spondylitis Disease Activity Index; BASFI: Bath Ankylosing Spondylitis Functional Index; BMD: bone mineral density; BMI: Body Mass Index; CRP: C-reactive protein; IBD: inflammatory bowel disease; mSASSS: modified Stoke Ankylosing Spondylitis Spinal Score; NSAIDs: non-steroidal anti-inflammatory drugs; SD: standard deviation; UCOASMI: University of Cordoba Ankylosing Spondylitis Metrology Index.

## Data Availability

Data are available upon reasonable request.
